# New Insights into the Inter-Individual Variability in Perspective Taking

**DOI:** 10.3390/vision1010008

**Published:** 2017-01-03

**Authors:** Henryk Bukowski, Dana Samson

**Affiliations:** 1 Psychological Sciences Research Institute, Université catholique de Louvain, Louvain-La-Neuve 1348, Belgium; 2 Social, Cognitive and Affective Neuroscience Unit, Department of Basic Psychological Research and Research Methods, Faculty of Psychology, University of Vienna, Vienna 1010, Austria

**Keywords:** individual differences, perspective taking, theory of mind, social cognition, empathy

## Abstract

This study aimed to test whether individual differences in perspective taking could be explained with two underpinning cognitive dimensions: The ability to handle the conflict between our egocentric perspective and another person’s perspective and the relative attentional focus during processing on the egocentric perspective versus another person’s perspective. We conducted cluster analyses on 346 participants who completed a visual perspective-taking task assessing performance on these two cognitive dimensions. Individual differences were best reduced by forming four clusters, or profiles, of perspective-takers. This partition reflected a high heterogeneity along both dimensions. In addition, deconstructing the perspective-taking performance into two distinct cognitive dimensions better predicted participants’ self-reported everyday life perspective-taking tendencies. Altogether, considering attentional focus and conflict handling as two potential sources of variability allows forming a two-dimensional space that enriches our understanding of the individual differences in perspective taking.

## 1. Introduction

Adults are not all equally good at taking someone else’s perspective. More specifically, they are not equally good at decentring from their own perspective and inferring the emotions, goals, or beliefs of other people. Individual differences in perspective taking have been demonstrated through several methods. The first and predominant method is through questionnaires about how participants perceive themselves in terms of perspective-taking competence in everyday life (measured, for example, by the Cognitive empathy subscale of the Empathy Quotient [1]) or in terms of their tendencies to engage in perspective taking (measured for example by the Perspective-taking subscale of the Interpersonal Reactivity Index [2]). Individual differences on these questionnaires have been considered in relation to, for example, alexithymia [3], multilingualism [4], and a higher propensity to forgive [5]. The second method consists of perspective-taking tasks used to assess participants’ performance in terms of accuracy and speed. How successful participants are in these tasks has been considered in relation to, for example, their level of schizotypy [6], their proneness to guilt [7], but also the efficiency of general cognitive abilities such as language, working memory, and executive functions (e.g., [8,9]).

In the current study, we take a new approach by investigating what, within perspective taking, contributes to successful performance. More specifically, our aim was to examine the extent to which individual differences could be better characterized by directly assessing how different specific cognitive facets of perspective taking contribute to the overall task performance. We focused on two sets of cognitive processes directly linked to current and recent views of the cognitive processes underlying perspective taking. These sets of cognitive processes will be referred to as two cognitive dimensions along which we aim to obtain two distinct performance scores.

The first dimension refers to the ability to handle conflicting perspectives. During our social interactions, we often hold a different view to that of others. The consideration of other people’s point of view therefore often requires the suppression of interferences from our own point of view [10,11,12,13,14], a computation that is thought to be achieved by domain general executive processes [9,10,12,15,16]. Accordingly, it has been shown that perspective-taking performance decreases when individuals concurrently perform a task tapping into executive processes [15,17,18,19]. Furthermore, it has been shown that, in both children and adults, perspective-taking performance varies from one individual to another depending on the individual’s performance in working memory and inhibitory control tasks [8,9,18,20,21,22,23,24,25,26,27,28,29]. The ability to handle conflicting perspectives is thus one cognitive dimension in which individuals differ and may determine individuals’ overall perspective-taking abilities. However, this may not be the sole dimension.

Another cognitive dimension in which individuals’ perspective-taking skills may vary relates to the attentional focus during social interactions or when we think about others. This dimension captures the relative focus between our own point of view and other people’s point of view. It is often considered that our own perspective is more salient. For example, it is well known that objects associated with the self are preferred [30], better attended to [31,32,33], and better remembered [34,35]. Several models also posit that when we think about other people’s point of view we use our own point of view as an anchoring point and then adjust it [11,36]. This explains why it can be so difficult to inhibit one’s own conflicting perspective to consider other people’s point of view and why, in both children and adults, egocentric biases are so prevalent in perspective-taking tasks [10,37].

Recent findings show, however, that the self-perspective is not always necessarily more salient or getting in the way when children or adults watch or interact with other people. For example, there is accumulating evidence showing that, at least in some circumstances, we spontaneously track other people’s mental states such as their beliefs [38,39,40] or their visual experiences [41,42], even when these mental states conflict with our own mental states. Such spontaneous tracking has been found to be maintained when individuals perform a concurrent effortful task [15], suggesting that attention can be drawn effortlessly to other people’s mental states. The relative attentional focus to one’s own and someone else’s perspective can thus be variable from one context to another (e.g., [43]) but may also, in the same context, vary from one individual to another. Individual differences in perspective taking may therefore also be explained in terms of the attentional focus on the self-perspective relative to the other-perspective.

In sum, we suggest that the common one-dimensional view of perspective taking through which we categorize individuals as either good or bad perspective-takers is limited. We propose a more sophisticated account whereby individual differences could be described via two dimensions: the capacity to handle conflicting perspectives and the attentional focus to the self- versus the other-perspective. This multidimensional approach provides a better understanding of the origin of poor or good perspective taking. For instance, we could determine whether someone’s failure to suppress his/her interfering egocentric perspective originates from his/her poor executive capacities or from the high attentional focus on his/her egocentric perspective.

We first hypothesized that our proposed two-dimensional approach is a relevant and useful approach to describe individual differences in perspective taking. Support for this hypothesis would come from the finding that there are indeed significant individual differences in both dimensions. Secondly, we hypothesized that in comparison to the one-dimensional measure of perspective taking, the two proposed dimensions together provide more explanatory power to examine the link with other inter-personally relevant variables, such as the propensity to engage in perspective-taking.

In order to test these hypotheses, we used a visual perspective-taking (VPT) task developed by Samson and colleagues [41]. The task measures level 1 VPT, which is the ability to infer which objects someone else can or cannot see (in contrast to the level 2 VPT that allows one to infer that an object may have a different appearance to someone else; [44]). In daily life, this basic form of VPT often serves as the basis for more sophisticated inferences, since what someone else is looking at gives us useful information about what that person wants, knows, thinks, or talks about. Variability in performance on this VPT task is thus relevant for perspective taking in everyday life.

**Figure 1:**
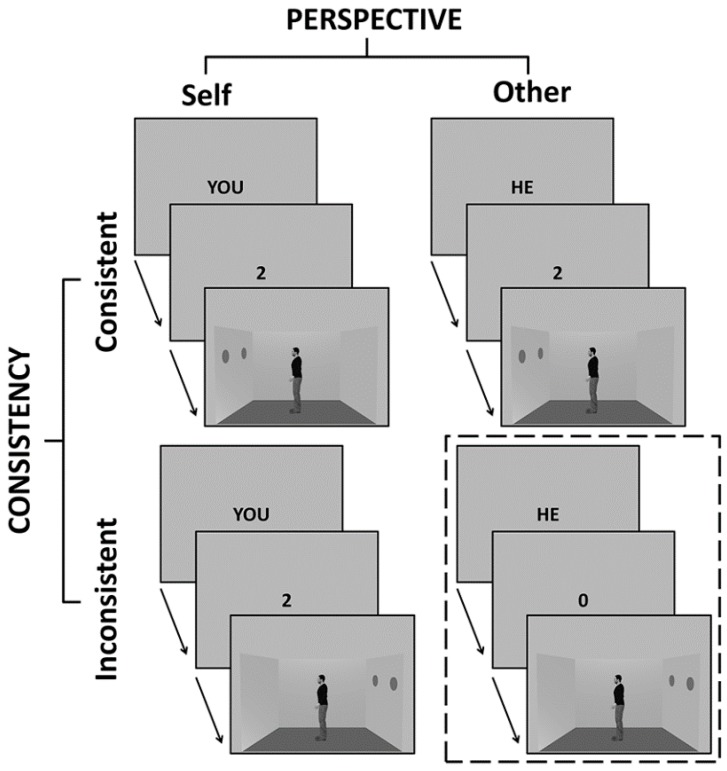
Illustrations of trials used in the VPT task across the four different experimental conditions. Participants were instructed to judge whether a number cue (ranging from 0 to 3) matched the number of dots visible from the prompted perspective, which could be either the participants’ perspective (self-perspective condition) or the perspective of another person in the room (other-perspective condition). The participants and the other person could see either a different number of dots (inconsistent condition) or the same number of dots (consistent condition). The common one-dimensional measure of perspective-taking is the ability to inhibit our egocentric perspective to correctly consider the other person’s differing perspective, which was captured here by the performance in the other-perspective/inconsistent-perspectives condition (dashed rectangle). The two hypothesized dimensions, the handling of conflicting perspectives, and the relative attentional focus on the self- versus other-perspective were measured by comparing performance on inconsistent and consistent perspectives trials (Conflict index) and on other- and self-perspective trials (Focus index), respectively.

More concretely, in the VPT task (see Figure 1), participants were instructed to either judge the number of red dots that they could see in a scene from their own perspective (self-perspective condition) or the number of dots that another person present in the scene could see (other-perspective condition). In some trials, what the participants saw from their perspective differed from what the other person saw (some of the dots were not visible to the other person, which means that the self-perspective and the other-perspective were inconsistent). In other trials, the participants and the other person saw the same number of dots and thus the two perspectives were consistent. This task allows us to extract a common one-dimensional measure of perspective taking, named Single-dimension index, that is to say the performance in taking another person’s perspective that is inconsistent with our egocentric perspective. This first measure corresponds to participants’ performance in the other-perspective/inconsistent perspectives condition (see the dotted rectangle in Figure 1). Importantly, the orthogonal manipulation of, on the one hand, the consistency of participants’ perspective with the perspective of the person in the room and, on the other hand, the perspective to judge allows us to obtain two key measures: First, we can measure the ability to handle conflicting perspectives by comparing the performance between trials where the perspectives are inconsistent and trials where the perspectives are consistent, irrespective of the perspective that participants have to judge (Conflict index). Secondly, we can measure the relative attentional focus on the self- and the other-perspective during judgments by comparing the performance between trials where participants have to judge the other-perspective and trials where participants have to judge the self-perspective, irrespective of the level of conflict between the perspectives (Focus index). Thus, the Single-dimension index served as a proxy to assess the traditional single dimension of perspective taking, whereas the Conflict and Focus indexes served as proxies to assess the two novel dimensions of perspective taking.

Based on these measures, we ran two separate cluster analyses that determine the extent to which existing individual differences in perspective-taking performance will translate into the partition of all individuals into statistically distinct and meaningful groups of perspective-takers. The first cluster analysis was based on the one-dimensional measure, while the second analysis was based on the two-dimensional measures. Depending on the number of subgroups obtained and how they differ from each other, we can determine how many dimensions are needed to distinguish the different groups of perspective-takers and whether decomposing the one-dimensional measure into two separate measures of perspective taking (the Conflict and Focus indexes) gives us more information about the origin of the inter-individual variability than relying on a single score of perspective-taking performance. Participants also filled out a self-reported questionnaire assessing their propensity to engage in perspective taking so that we could assess the extent to which the one-dimensional and two-dimensional measures predicted the motivation to engage in perspective taking in everyday life.

**Figure 2:**
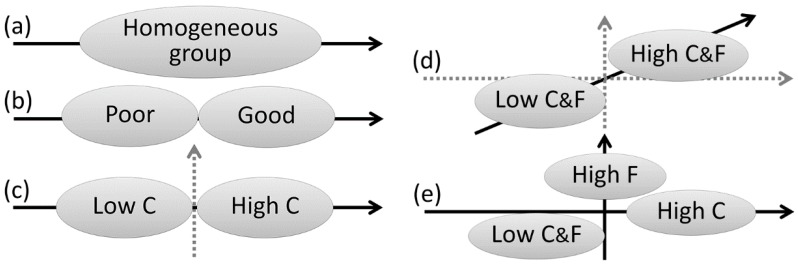
Five hypothetical outcomes regarding individuals’ partitioning into distinct groups of perspective-takers and the minimum number of dimensions needed to distinguish them. Individuals could form (**a**) a homogeneous group, or several groups that are distinguishable according to (**b**) the only dimension considered; (**c**) only one of the two dimensions considered; (**d**) a single dimension unspecific to any of the two dimensions; or (**e**) two dimensions. Black and grey arrows represent the hypothetical dimensions necessary and unnecessary, respectively, to distinguish the different groups of perspective-takers. C = Conflict index, F = Focus index.

For the single dimension cluster analysis, two outcomes were possible: the cluster analysis could result either in one group, meaning that there were not enough individual differences to distinguish individuals in distinct groups (see Figure 2a), or several distinct groups. This latter result would indicate that significant individual differences exist in our sample. However, these subgroups would only be distinguishable along a one-dimensional continuum, where some individuals could be classified as good perspective-takers and others as poor perspective-takers (Figure 2b).

If there is enough inter-individual variability with the one-dimensional measure in our sample, we can then test whether these individual differences can be decomposed into distinct sources of variability, that is to say, into distinct dimensions in which individuals vary. For the two-dimensional cluster analysis, three outcomes were possible.

One possible outcome is that the different groups of perspective-takers can be fully distinguished along only one of the two indexes introduced in the analyses, which would indicate that one of our indexes does not reflect a dimension in which individuals vary. If this result is found, we would not be in a position to demonstrate that a two-dimensional assessment is more informative than a one-dimensional assessment. Given the evidence reviewed earlier, if only one of the two measures captures inter-individual variability, it would most likely be the Conflict index, which taps into individuals’ ability to handle conflicting perspectives (see Figure 2c).

A second possibility is that the different groups of perspective-takers can be distinguished along both measures but in a highly similar fashion. For example, individuals with a high or low Conflict index would have, respectively, a high or low Focus index. This high interdependence between the two indexes would indicate that the different groups can actually be fully distinguished with only one of the indexes or another dimension that summarizes the two indexes (see Figure 2d). Following such an outcome, we would again be unable to demonstrate that a two-dimensional assessment is more informative than a one-dimensional assessment.

Finally, the third possibility is that the different groups of perspective-takers can be best distinguished when the two indexes are used (see Figure 2e). This would indicate that each index captures a unique extent of inter-individual variability and that their combination can form a meaningful two-dimensional space that provides a better and richer assessment of inter-individual variability in perspective-taking performance.

In order to run reliable cluster analyses, we needed a large sample of participants who had completed the VPT task. To this end, we merged the datasets from six different experiments to form a sample of 346 participants who all completed the VPT task (see Table 1). Some of these experiments included conditions in which emotional states (guilt, anger, shame, sadness, and happiness) were induced. A few studies have started to highlight the impact of emotions on perspective-taking performance [45,46,47]. The inclusion of these conditions thus reflects a natural source of variability in perspective taking that individuals can encounter over the course of a single day. Finally, in order to replicate our findings, the cluster analyses were also run on an additional dataset of 260 participants.

To summarize, we propose a novel approach to asses individual differences in perspective taking by measuring performance along two cognitive dimensions underpinning perspective-taking. We tested through a cluster analysis whether there are enough individual differences in these two dimensions to justify their use and we tested through a regression analysis whether these novel dimensions have a better predictive power of self-reported everyday life perspective-taking abilities than a typical one-dimensional measure.

## 2. Results

### 2.1. One-Dimensional Cluster Analysis

**Figure 3:**
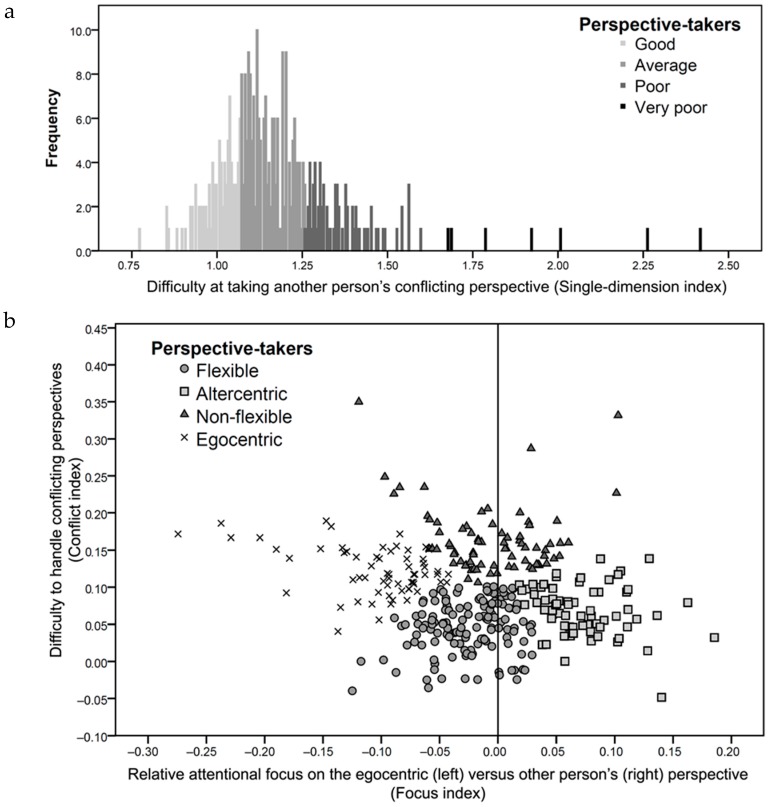
(**a**) One-dimensional clustering of participants’ difficulty in considering another person’s differing perspective (Single-dimension index); (**b**) Two-dimensional clustering of participants’ perspective-taking performance based on their difficulty in handling conflicting perspectives (Conflict index) and the attentional focus on the other person’s perspective versus the self-perspective (Focus index).

When introducing in the cluster analysis participants’ performance at taking another person’s conflicting perspective (Single-dimension index) as a unitary construct to assess perspective-taking inter-individual variability, we obtained a four-group partition (see Table 2 for group centroid characteristics). This partition allows us to distinguish individuals only along a one-dimensional continuum on which the four groups can be characterized by either having good (*N* = 92), average (*N* = 177), poor (*N* = 70), or very poor (*N* = 7) perspective-taking performance (see Figure 3a).

### 2.2. Two-Dimensional Cluster Analysis

When introducing in the cluster analysis participants’ performance at handling conflicting perspectives (Conflict index) and the relative attentional focus between the self- and other perspectives (Focus index) as two dimensions to assess inter-individual variability in perspective taking, we obtained a four-group partition (see Table 2 for group centroid characteristics and Figure 3b for the distribution of individuals according to the two dimensions). The same partition was obtained whether the Bayesian or the Akaike’s information criterion was used to determine the number of clusters that best explained the observed variability. We can note that if we consider only the Consistency index, only two of the four groups can be distinguished. These two groups are characterized by having either high or low values on the Consistency index, which indicates that the two groups had more or less difficulty in handling conflicting perspectives. We labelled these two groups “non-flexible” and “flexible” perspective-takers, respectively. Critically, the two remaining groups could only be distinguished by the Focus index. They were characterized by having either highly negative or highly positive values on the Focus index, which indicates that the two groups focused on either the self- or the other-perspective, respectively. We labelled these two groups as “egocentric” and “altercentric” perspective-takers, respectively. This result shows for the first time that there is a large amount of inter-individual variability regarding the relative attentional focus on the egocentric versus the other people’s perspective [49] and that not all individuals focused on their egocentric perspective (20% even show the reverse). It is important to note, however, that, on average, individuals have a negative Focus index (*M* = −0.0161, *SD* = 0.067), which means that, in line with most perspective-taking studies, individuals are, on average, significantly better at taking their egocentric perspective over another person’s perspective, *t* (345) = 4.443, *p* < 0.001 [50].

Finally, the same two-dimensional cluster analysis was conducted on an additional dataset of 260 individuals, which resulted in a similar four-cluster partition. The groups of perspective-takers were only distinguishable when both dimensions were considered, which replicates our finding that there are significant individual differences on both dimensions (see Supplementary Materials Section 2).

### 2.3. Predicting Self-Reported Everyday Life Perspective-Taking Propensity with One-Dimensional versus Two-Dimensional Measures

**Figure 4:**
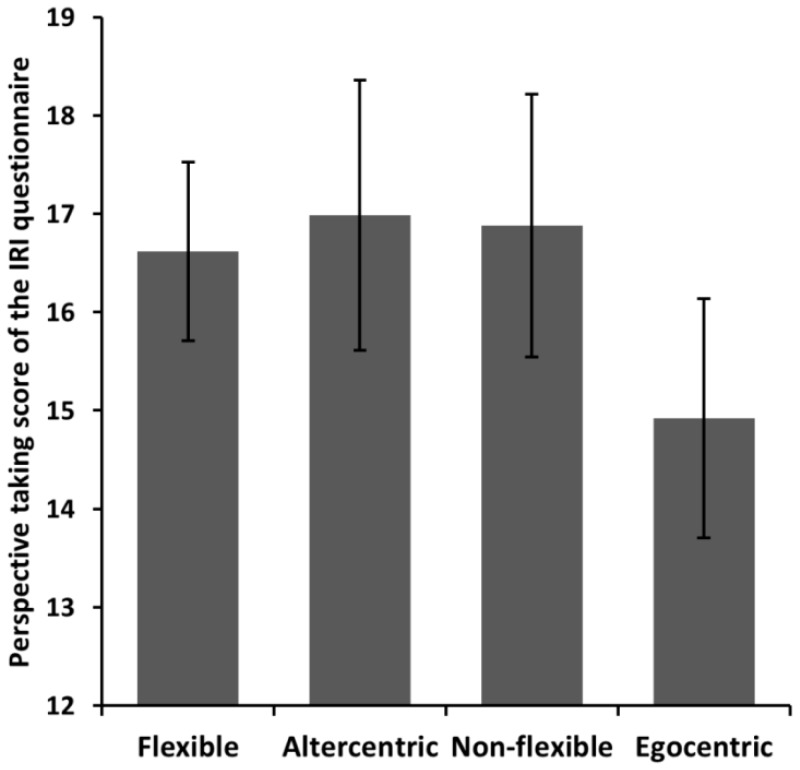
Perspective-taking scores measured by the IRI questionnaire in function of the groups of perspective-takers. Error bars represent 95% confidence interval.

The regression analysis showed that participants’ Single-dimension index only marginally predicted their PT-IRI scores, β = −0.108, *t* (282) = 1.826, *p* = 0.069, which explained 1.2% of the variance (*R*^2^ = 0.012). In contrast, the combination of the Conflict and the Focus indexes explained 3.3% of the variance (*R*^2^ = 0.033, *F* (2, 282) = 4.770, *p* = 0.009). Participants’ PT-IRI scores were not significantly predicted by participants’ Conflict index, β = 0.021, *t* (282) < 1, *p* = 0.728. Participants’ Focus index, however, significantly predicted participants’ PT-IRI scores, β = 0.184, *t* (282) = 3.084, *p* = 0.002. In other words, higher attentional focus given by individuals to their egocentric perspective compared to other person’s perspective in the VPT task significantly predicted a lower perspective-taking propensity on the IRI questionnaire (see also Figure 4 for a comparison of perspective-taking scores across the four profiles of perspective-takers). Importantly, in a separate regression analysis with PT-IRI as the dependent variable, adding the Focus and Conflict indexes to the model initially containing the Single-dimension index significantly improved the model, increasing the total explained variance from 1.2% to 3.4%, *F* change (2, 279) = 3.278, *p* = 0.042, which indicates that shifting from a one-dimensional to a two-dimensional model led to a significant improvement in predicting the PT-IRI scores.

Thus, decomposing the common one-dimensional measure of perspective-taking into two distinct dimensions allowed for discovering a significant predictor of real-life perspective-taking propensity.

### 2.4. Comparison of the One-Dimensional and Two-Dimensional Profiles

**Figure 5:**
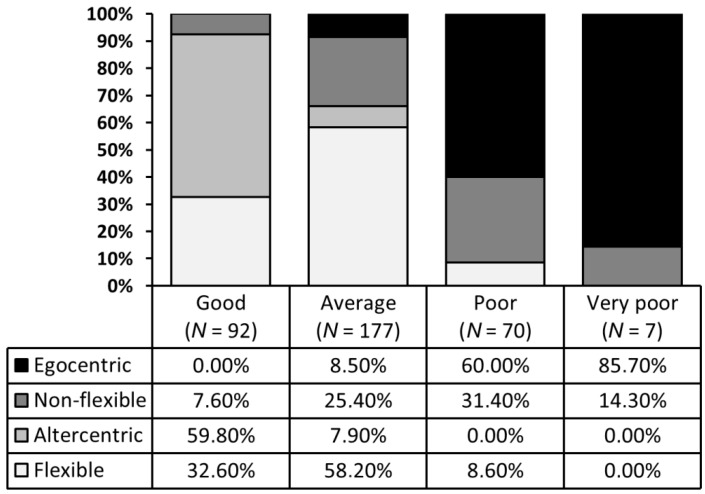
Distribution of profiles of perspective-takers between the two-dimensional and one-dimensional partition.

The Pearson’s chi-square analysis revealed a highly significant rejection of the null hypothesis of independence between the one-dimensional (“good” to “very poor” perspective-takers) and two-dimensional partitions of participants, χ*^2^* (9, *N* = 346) = 260.680, *p* < 0.001. When plotting how each profile of perspective-takers of the one-dimensional partition is distributed across each profile of perspective-takers of the two-dimensional partition (see Figure 5) we can note that the two partitions do not overlap. Instead, we can see that the “good” perspective-takers profile can be broken down into individuals who have little difficulty in either handling conflicting perspectives or prioritizing another person’s perspective over the self-perspective. As for the “poor” perspective-takers, they can be broken down into individuals who have great difficulties in handling conflicting perspectives or prioritizing their egocentric perspective over the other person’s perspective.

### 2.5. Distribution of Profiles across Emotion Induction Conditions

**Figure 6:**
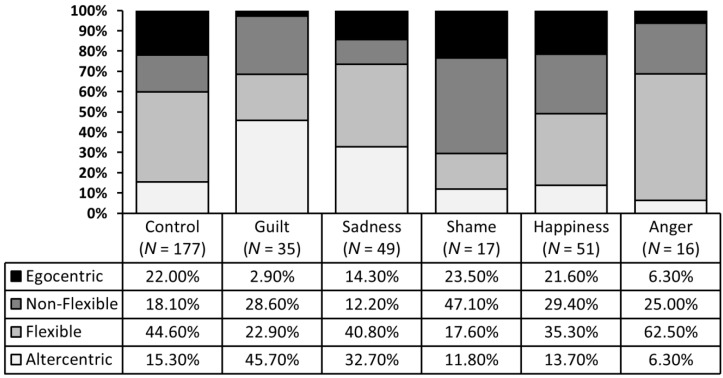
Distribution of the two-dimensional profiles of perspective-takers within the emotion conditions.

The Pearson’s chi-square analysis revealed a highly significant rejection of the null hypothesis of independence in the distribution of each profile of perspective-takers across the different emotion induction conditions, χ^2^ (15, *N* = 345) = 47.06 6, *p* < 0.001, which suggests that participants’ perspective-taking performance can be highly influenced by transient factors such as their emotional state (see Figure 6). For example, while one-sixth of the 177 participants included in a control condition were classified as non-flexible or altercentric perspective-takers, twice this proportion was found in the guilt and shame conditions, respectively (see [45] for detailed results on these effects of guilt and shame on perspective-taking).

## 3. Discussion

An individual’s performance in a perspective-taking task is usually graded along a one-dimensional continuum spanning from poor to good perspective taking. This study showed, however, that inter-individual variability taken from a sample of 346 healthy individuals could be better captured by decomposing the common one-dimensional measure of perspective-taking into two cognitive dimensions likely shared across all forms of perspective-taking: the ability to handle conflicting perspectives and the relative attentional focus on the egocentric perspective versus other people’s perspectives. These two cognitive dimensions were not redundant in explaining inter-individual variability because individuals could score high or low on one dimension but not on the other and because the cluster division obtained by taking into account two dimensions did not overlap with the cluster division obtained with a common one-dimensional perspective-taking measure. Furthermore, we found that deconstructing the perspective-taking performance into two dimensions allowed us to better predict individuals’ self-reported perspective-taking propensity in daily life (as assessed by the IRI questionnaire). The significantly increased (almost tripled) predictive power came from the fact that, between the two dimensions, the relative attentional focus that is given to the self- versus the other-perspective had a strong predictive power. This finding shows that assessing the two dimensions separately allows for testing and capturing effects that may have been not captured with the traditional one-dimensional measure of perspective-taking performance.

Participants’ partitioning into two opposite groups regarding their ability to handle conflicting perspectives clearly indicates that there exists a large amount of inter-individual variability in this dimension. Many studies have highlighted the role of domain general executive abilities in the ability to overcome the conflict between the egocentric perspective and another person’s perspective [15,21]. For example, young children or adults with frontal brain damage show poor performance on classic perspective-taking tasks but reducing the level of conflict between perspectives significantly increases their level of performance [22,51,52,53,54]. Thus, given the known inter-individual variability observed in measures of domain general executive abilities it is not surprising to have found that the ability to handle conflicting perspectives is one dimension that explains individual differences in perspective-taking performance. However, individuals’ performance on executive tasks cannot entirely predict their performance on perspective-taking tasks (e.g., [13,23]) and several studies in infants suggest that it is possible to correctly infer another person’s conflicting perspective despite a reduced ability to handle conflicting perspectives [38,55,56,57]. This is why we expected that at least one additional dimension must underpin perspective-taking performance.

Among the four groups of perspective-takers we obtained, only two could be distinguished in terms of the difficulty of handling conflicting perspectives. The two other groups could be distinguished when another dimension was considered: the attentional focus on the egocentric perspective relative to other people’s perspective during processing. This means that there was enough variability along this dimension to form two opposite groups of perspective-takers. One-fifth of our participants were characterized by a higher attentional focus on the other person’s perspective than their egocentric perspective. This finding qualifies the widespread view that the egocentric perspective is always prioritized, accessed first, and used as an anchoring point during perspective taking [10,11,36,58,59], since for some individuals this was not the case. This finding is, however, consistent with recent studies showing that, in some situations, another person’s perspective receives at least as much attention as the egocentric perspective. For example, holding a conflicting egocentric perspective did not prevent adults from spontaneously tracking other people’s mental states [38,39,40,41,42], even when they concurrently performed an effortful executive task [15]. Furthermore, some studies have shown that, in the absence of conflict between perspectives, adults judged faster what another person saw than what they saw from their egocentric perspective [15,37,41,42,60]. Together, these studies suggest that the egocentric perspective is not always as prioritized as usually assumed. In this study, we show for the first time that the extent of this prioritization differs across individuals and therefore that prioritization of our egocentric perspective should not be considered a universal characteristic. Interestingly, of the two dimensions, attentional focus may be the one most critically linked to the spontaneous tracking of other people’s mental states. The observation that individuals differ from one another on this dimension then also suggests that there might be important individual differences in the ability to spontaneously track other people’s mental states in daily life. Findings of spontanenous or automatic mental state tracking may thus not be as universal as previously thought.

Together, the four groups, or profiles, of perspective-takers seem to reflect a high heterogeneity in the adult population, where individuals with poor conflict-handling skills and high egocentrism would have otherwise been merged into a single group of “poor” perspective-takers, and individuals with a high altercentrism and good conflict-handling skills would have been merged into the “good” perspective-takers group (see Figure 4). Therefore, considering the attentional focus given to the egocentric perspective versus the other person’s perspective as a potential source of variability allows us to form, in combination with the ability to handle conflicting perspectives, a two-dimensional space that leads to a richer understanding of the underpinning causes of individual differences in perspective taking.

We found that one dimension—the attentional focus given to the egocentric perspective relative to the other person’s perspective—significantly predicted participants’ perspective-taking propensity. More specifically, the participants characterized as highly focused on the self (i.e., the egocentric group) were those who reported a significantly lower propensity to engage in perspective taking in their everyday life than all other groups of perspective-takers (see Figure 4). This suggests that motivational factors are more related to the deployment of attention to the self versus the other than to the recruitment of cognitive resources to handle conflicting perspectives. The fact that the traditional one-dimensional measure of perspective taking indiscriminately assesses both the relative attentional focus between perspectives and the capacity to handling conflicting perspectives thus explains the smaller predictive power of this unidimensional measure on perspective-taking propensity.

Importantly, the fact that only one dimension predicted perspective-taking tendencies does not indicate that one dimension is enough to predict individual differences in perspective-taking; the predictive value of the ability to handle conflicting perspectives has been largely documented when the ability rather than the motivation to take someone else’s perspective was measured [9,18,21]. Because the perspective-taking subscale of the IRI (Davis, 1980) is geared towards the motivational aspects, it is possible that the Consistency index would be associated with scores on a real-life self-reporting measure of perspective taking if we had used another questionnaire, more geared toward actual abilities, such as the cognitive empathy subscale of the Empathy Quotient [1,61]. In other words, there is no doubt that successful perspective taking in real life also depends on the ability to handle conflicting perspectives and therefore both dimensions are necessary. People have different knowledge and experiences of the world and therefore adequate perspective taking almost always requires handling conflicting perspectives.

It may seem that, with the VPT task, by orthogonally crossing the perspective to take and the level of conflict between perspectives, we can assess the conflict-handling ability and the relative self–other perspective focus as two independent dimensions. It should be clarified that these two dimensions are not independent; they are underpinned by different cognitive mechanisms that strongly interact. The ease in taking another person’s perspective depends on how salient the information pertaining to the other person’s perspective is. However, this salience can be voluntarily biased through top-down influences in the attempt to resolve a conflict between perspectives. Conversely, the salience of one perspective directly impacts on the performance in inhibiting this perspective. In sum, while these two dimensions can be independently measured, they relate to cognitive processes that are jointly deployed and interact during perspective taking.

Across the variety of emotion induction conditions participants were allocated to, we found very different proportions of egocentric, altercentric, non-flexible, and flexible perspective-takers. For example, we found that the proportion of altercentric and non-flexible perspective-takers was twice as high in the guilt and shame induction conditions, respectively, than in the control conditions. This finding suggests that our emotional state may affect our perspective-taking performance, which is in line with prevous studies [46,47] that reported a beneficial influence of sadness and guilt and a detrimental influence of shame and happiness. Because our emotions change over the course of a single day, this finding highlights the importance of considering perspective taking as a dynamic ability rather than a trait-like, or static, characteristic.

All our findings are based on a single task that measures level 1 VPT. This basic form of VPT has been proposed to be achieved via the computation of the other person’s line of sight and is considered as a building block of theory of mind development [62,63,64,65]. However, one might wonder whether our findings can be generalized to the other forms of perspective taking (e.g., when beliefs or desires must be inferred) present in real life and in other perspective-taking tasks. The most direct evidence regarding this issue is that we found that participants’ relative attentional focus on the self- versus other-perspective significantly predicted participants’ self-reports of perspective-taking tendencies in their everyday life. This suggests that the performance on the VPT task we used reflects to some extent a real-life facet of perspective taking. Furthermore, the observed effects of guilt, shame, and sadness on the VPT that we used are congruent with those observed on other perspective-taking tasks in other studies [7,46,47]. It seems, therefore, reasonable to think that, although based on a measure of a simple form of perspective taking, our findings can be generalized to perspective taking in real life and as studied on other tasks. However, there are likely to be other dimensions of perspective taking currently hidden in more complex perspective-taking tasks that could, perhaps, also be unpacked and complement the multidimensional approach.

For the sake of clarity, it is important to underline that, although we replicated our initial four-cluster partition with a different sample of healthy individuals who performed the same visual perspective-taking task, we do not necessarily expect to find the exact same partitioning in other samples or in other forms of perspective taking (for example, the number of clusters could differ). The important finding that should be generalized, however, is that there exist important individual differences along at least two cognitive dimensions and that the cluster partitions should result in groups that are articulated along both dimensions.

Finally, the current study aimed to show the extent of potential inter-individual variability within a relatively homogeneous population (i.e., university students) across diverse emotion conditions (see Figure 3) and control conditions (see Supplementary Materials Figure S1). For this reason, our sample of participants is not, and was never meant to be, representative of the general population. Nevertheless, because the general population is much more heterogeneous, more individual differences in perspective taking are expected in this population. We are, therefore, confident that the two-dimensional variability we reported here can be generalized to the general population.

## 4. Materials and Methods

### 4.1. Participants

We merged the data from three studies conducted in our research group, totalling six experiments with a sample size of 346 healthy individuals (59% females, mean age: 21.5, age range: 18–33). All volunteers participated in return for course credits or 8–16 euros. Approximately half of the participants (*N* = 164) were in an emotion induction condition. All experiments were conducted at the Université Catholique de Louvain and were approved by the ethics committee of the Psychological Sciences Research Institute (Projet 2012-27, November 2012).

### 4.2. Instruments

#### 4.2.1. Visual Perspective-Taking Task

Perspective-taking performance was measured by the VPT task [41,66,67]. Participants saw a picture of a room with a human model positioned in the centre and red dots pinned on one or both side walls (see Figure 1). The human model was shown in profile, facing either the right or the left wall. Prior to the presentation of the visual scene, a perspective prompt indicating which perspective to take (“YOU” or “SHE”/”HE”) and a number prompt (ranging from 0 to 3) indicating a number of dots to verify were presented. Participants were asked to judge whether the number prompt matched the number of dots visible from either the participant’s perspective (self-perspective condition) or from the human model’s perspective (other-perspective condition) by pressing the upward arrow (yes) or downward arrow key (no). For example, after the prompts “HE” and “2”, participants had to judge whether the model could see two dots in front of him. The number of dots visible could be different between the two perspectives (inconsistent perspectives condition) or identical for both perspectives (consistent perspectives condition). Directly after the participant’s response, a feedback “Correct”, “Incorrect”, or “No response” was presented. A “No response” feedback was presented after 2 s had elapsed without a response from the participant. Errors and reaction times were collected. The task was run with the same timing of events as in the original study (see [41], for a detailed description of the task) but with E-prime (Psychology Software Tools, Pittsburgh, PA, USA).

In Experiments 1, 2, and 3, like in the original paradigm, there were 24 matching trials and 24 mismatching trials in each of the four experimental conditions (2 (Perspective: self vs. other) × 2 (Consistency: consistent vs. inconsistent)). In addition, 16 filler trials were included to avoid anticipatory responses (see the original study for details). Furthermore, because mismatching trials in the consistent condition displayed number prompts irrelevant for any perspective and therefore were particularly easy to process, mismatching trials were unbalanced in terms of performance difficulty compared to matching trials and therefore were not analysed (this was also the case in the original study). The task included a total of 234 trials divided into four blocks of 52 trials plus a set of 26 practice trials. Trials within each block were presented in a randomized order. In Experiments 4, 5, and 6, we used a shortened version of the VPT task, with two blocks instead of four, in which the findings of the original paradigm were replicated, suggesting that the shorter version was also suited to measure VPT performance. The human model was a female confederate student in Experiment 1, 2, and 3, and a gender-congruent human avatar in Experiments 4, 5, and 6. Further details on the experiments can be found in the Supplementary Materials Section 1 and in [45,48].

#### 4.2.2. Perspective-Taking Propensity

We measured self-reported perspective-taking propensity with the Interpersonal Reactivity Index (IRI [2]), a questionnaire measuring participants’ agreement on a five-point Likert scale with 28 statements about their habits, beliefs, and experiences in various social and emotional situations. The IRI is divided into four subscales: perspective-taking, fantasy (i.e., self-absorption in fictions), empathic concern, and personal distress. For example, one of the seven items of the perspective-taking subscale states “I sometimes try to understand my friends better by imagining how things look from their perspective”. As we were specifically interested in measuring perspective taking, we only analysed the score on the perspective-taking subscale, labelled PT-IRI score, which could go from 0 to 28. The IRI was completed prior to the emotion induction (when there was one) by all participants, except for the participants of Experiment 4, who did not complete the IRI and one participant of Experiment 6 whose data was lost due to a technical failure. Overall, 283 participants completed the IRI.

### 4.3. Data Analyses

#### 4.3.1. Indexes Computation

In order to obtain normalized measures of one-dimensional and two-dimensional measures of perspective-taking, we calculated three indexes: the Single-dimension index, the Focus index, and the Conflict index. The Single-dimension index is a one-dimensional measure of the ability to consider another person’s differing perspective, while the Conflict and the Focus indexes measure the ability to handle conflicting perspectives and the relative attentional focus on the self versus the other person’s perspective, respectively.

To calculate these indexes, we first computed participants’ reaction times (RT) for correct responses and error rates (ER) in the VPT task. Then, in order to obtain a single measure of performance per dimension (and to homogenise the potentially different speed–accuracy trade-offs across participants), we merged the RT for correct responses and ER into inverse efficiency scores (IES = RT/(1 − ER) [68]). Some cautions have been expressed about the use of IES [69]: It is recommended not to use the IES if the speed–accuracy trade-offs are too important, which is indicated by a lack of positive correlation between RT and ER. In our dataset, RT and ER on the visual perspective-taking task were positively correlated, *r* (346) = 0.228, *p* < 0.001. In addition, it is recommended not to use the IES if the average ER exceeds .10 because the IES presents the disadvantage that the RTs are non-linearly multiplied as the ERs increase. The mean ERs were lower than 0.10 in all experiments. Therefore, both recommendations about the use of the IES were satisfied.

The Single-dimension index was calculated from participants’ IES in the other-perspective/inconsistent perspectives condition of the VPT task (see the dotted rectangle in Figure 1), with a higher value indicating lower (one-dimensional) perspective-taking performance, or a higher egocentric bias. The Conflict index was calculated from the subtraction of participants’ IES in the consistent perspectives condition from their IES in the inconsistent perspectives condition, with a higher value indicating more difficulties in handling conflicting perspectives. The Focus index was calculated from the subtraction of participants’ IES in the other-perspective condition from their IES in the self-perspective condition, with a positive value indicating better performance in taking the other person’s perspective than the self-perspective. Finally, to normalize the three indexes in terms of unspecific global response speed and accuracy, each index was divided by the participant’s global IES (i.e., with all four conditions merged). Altogether, these transformations allowed to obtain, for each participant, three indexes of perspective-taking performance that specifically captured the perspective-taking dimensions of interest and not participants’ general speed or accuracy, which are unrelated to perspective taking itself.

#### 4.3.2. Cluster Analyses

Building on recent studies suggesting that there are (at least) two dimensions underpinning perspective-taking performance, our main hypothesis was that individuals will significantly differ from each other and in both dimensions. Support for this hypothesis would come from the finding that individuals cluster in meaningful groups, or profiles, of perspective-takers distinguishable only in a two-dimensional space. In order to explore how individuals cluster on the basis of their perspective-taking performance, we ran two two-step cluster analyses: the first with the Single-dimension index and the second with the Conflict and the Focus indexes. Clustering consists of partitioning cases (here individuals) into groups that minimize within-group variability and maximize between-group variability [70]. The two-step technique consists of first forming small sub-clusters of individuals to reduce the size of the matrix and then progressively merging them following a hierarchical clustering algorithm. We chose this two-step technique because it offers the advantage that no arbitrary prescribed number of clusters is needed. The obtained final partition is the one that minimizes the Bayesian or Akaike’s information criterion, two widely used statistical indexes for model selection that measure the efficiency of a model in predicting the data while penalizing the complexity of the model [71,72].

To address some concerns that the Focus and Conflict indexes introduced in the cluster analysis measure only the main effects but not their interaction, we ran an additional cluster analysis with four indexes, each representing the performance in each experimental condition (con-other, incon-other, con-self, incon-self). As a result, an almost identical two-dimensional partition was obtained (see Supplementary Materials Section 2, Table S1).

Finally, for the sake of reliability, the two-dimensional cluster analysis was conducted on an additional dataset of 260 participants obtained through merging the samples from four experiments (described in Supplementary Materials Section 3, Tables S2 and S3).

#### 4.3.3. Regression Analyses

It was also hypothesized that adopting the two-dimensional approach can be more useful than using the common one-dimensional measure of perspective taking when trying to relate the measured task performance with self-reported measures of perspective-taking ability in everyday life. More specifically, we tested whether the Conflict index and/or the Focus index are better associated with the self-reported propensity to engage in everyday perspective-taking than the Single-dimension index. For this purpose, we compared two regressions analyses on the PT-IRI scores: One with the Single-dimension index for predictor, and the other with the Conflict and the Focus indexes as predictors. We also ran an additional regression with the Perspective × Consistency interaction factor, but this interaction factor had no significant predictive power (see Supplementary Materials Section 4).

#### 4.3.4. Tests of Independence

We investigated whether the different profiles of perspective-takers obtained through the two-dimensional cluster analysis were equally distributed across (1) the different profiles obtained through the one-dimensional cluster analysis and (2) the different emotion induction conditions (control, guilt, sadness, shame, happiness, and anger). To do so, we ran two chi-square tests of independence between the two-dimensional cluster membership and either the one-dimensional membership or the emotion induction condition membership.

## 5. Conclusions

It is widely assumed that relying on our egocentric perspective is a universal prepotent tendency when inferring other people’s mental states, which led researchers to view perspective taking as resulting from the single ability to suppress or correct this egocentric tendency [10,11,12,13]. This assumption is reflected in the fact that existing tasks often assess perspective-taking performance along a one-dimensional continuum. We analysed the perspective-taking performance of 346 healthy adults on a task that separately assessed the relative attention focus on the egocentric perspective versus the other person’s perspective and the ability to handle conflicts between perspectives. We found a high heterogeneity along both dimensions that gave a richer account of the source of individual differences underpinning perspective taking than a one-dimensional continuum. Perspective-takers that would typically be characterized as “good” were either individuals who efficiently handled conflicts or strongly focused on the other person’s perspective instead of their own perspective. On the other hand, perspective-takers that would typically be characterized as “poor” were individuals who either had more difficulty in handling conflicting perspectives or strongly focused on their egocentric perspective. Furthermore, while both the ability to handle conflicting perspectives and the attentional focus on the self versus others explained the variability in the perspective-taking task, only the latter significantly predicted self-reported propensity to engage in perspective taking in daily life. This study thus paves the way for a multidimensional approach to the study of individual differences in perspective taking. Such a multidimensional approach provides a much richer theoretical background and a practical tool to understand individual differences in perspective taking.

## Figures and Tables

**Table 1:** Description of the merged dataset.

Exp	Conditions (*N*)	% F	*M* Age	Reference
1	Guilt (17), Anger (16), Control (17)	52.9	21.5	[45]
2	Guilt (18)	50.0	21.2	[45]
3	Shame (17), Control (17)	55.9	21.0	[45]
4	Sadness (19), Happiness (22), Control (21)	67.7	20.4	[48]
5	Sadness (30), Happiness (29), Control (29)	87.5	21.5	[48]
6	Control (93)	32.3	22.5	[48]

**Table 2:** Description of the two cluster partitions based first on participants’ difficulty in considering another person’s differing perspective (Single-dimension) and, secondly, on both the difficulty in handling conflicting perspectives (Conflict) and the attentional focus on the other person’s perspective relative to the self-perspective (Focus).

**Source Variables**	**Single-Dimension Index**			
Cluster #	1	2	3	4
Label	Average	Good	Poor	Very poor
*N* (% total)	177 (51.2%)	92 (26.6%)	70 (20.2%)	7 (2.0%)
Female %	71.4%	62.9%	56.5%	58.2%
Single-dim. *M* (*SD*)	1.153 (0.055)	0.999 (0.057)	1.367 (0.885)	1.965 (0.286)
**Source Variables**	**Conflict and Focus Indexes**			
Cluster #	1	2	3	4
Label	Flexible	Non-flexible	Altercentric	Egocentric
*N* (% total)	139 (40.2%)	75 (21.7%)	69 (19.9%)	63 (18.2%)
Female %	55.4%	62.7%	59.4%	61.9%
Conflict *M* (*SD*)	0.046 (0.034)	0.164 (0.045)	0.069 (0.034)	0.122 (0.032)
Focus *M* (*SD*)	−0.026 (0.032)	−0.005 (0.042)	0.073 (0.036)	−0.105 (0.048)
Single-dim. *M* (*SD*)	1.12 (0.076)	1.228 (0.164)	1.006 (0.078)	1.399 (0.212)

## Supplementary Materials

The supplementary materials are available online at www.mdpi.com/2411-5150/1/1/8/s1.
