# Supplementary material for: New Insights into the Inter-Individual Variability in Perspective Taking
Source: Vision (Basel). 2017 Jan 3;1(1):8. doi: 10.3390/vision1010008 (PMC6835961; doi:10.3390/vision1010008)
Supplement: Supplementary file 1 [file vision-01-00008-s001.pdf]

Supplementary Materials: New Insights into the Inter-Individual Variability in Perspective Taking

Henryk Bukowski and Dana Samson

Section 1: Description of the Experiments of the First (Main) Dataset

Experiments 1, 2, and 3 are fully described in [1] but can be summarized as follows: Participants were lead to believe that they played an interactive game on a computer with another person in order to induce contrasting emotional feelings, after which they completed the VPT task. The pictures of the human agent of the task were pictures of the other person. The interactive game was manipulated to elicit feelings of guilt (the other person lost money because of the participant), anger (the participant lost money because of the other person who refused to share some of his money), and shame (no money was involved but the participant was clearly less competent than the other person). Before the end of the experiment, participants completed an exit questionnaire assessing how they felt about the interactive game. The emotional inductions were successful except for some participants who were replaced.

Experiments 4 and 5 are fully described in [2] but can be summarized as follows: In Experiment 4, before completing either the VPT task or a False Belief task adapted from [3], participants watched a video clips inducing a sad, happy, or neutral mood. Then, after completion of either the VPT or False Belief task, participants recalled a personal event congruent with the emotional tone of the video clip and completed the other PT task. Before the end of the experiment, participants completed an exit questionnaire assessing how they felt after watching the video clip and recalling the personal event. The emotional inductions were successful. Experiment 5 was similar to Experiment 4 except that the False Belief task was replaced by a gaze cueing task (as in Experiment 1 of [4]).

Experiment 6 is fully described in [2] but can be summarized as follows: After completing the consent forms, participants completed the Positive and Negative Affects Scale [5] to assess their initial mood state. Then, they read the instructions related to the VPT task and completed the task.

Section 2: Cluster Analysis Results with Four Indexes of Perspective Taking

Table S1. Cluster analysis results with four indexes of perspective taking.

Source Variables	Con-Other, Inc-Other, Con-Self, Inc-Self			
Cluster #	1	2	3	4
Label	Flexible	Non-flexible	Altercentric	Egocentric
N (% total)	142 (41.2%)	82 (23.7%)	56 (16.2%)	63 (18.2%)
Female %	56.3%	67.1%	53.6%	59.1%
Con-Other M (SD)	0.947 (0.052)	0.84 (0.052)	0.847 (0.054)	0.888 (0.07)
Inc-Other M (SD)	1.11 (0.08)	1.186 (0.095)	1.008 (0.081)	1.426 (0.229)
Con-Self M (SD)	0.965 (0.057)	0.878 (0.062)	1.034 (0.061)	0.866 (0.07)
Inc-Self M (SD)	0.988 (0.082)	1.15 (0.126)	1.141 (0.091)	0.937 (0.079)
Consistency M (SD)	0.045 (0.033)	0.151 (0.04)	0.066 (0.034)	0.132 (0.047)
Perspective M (SD)	−0.024 (0.035)	0.003 (0.037)	0.08 (0.034)	−0.104 (0.047)

Section 3: Two-Dimensional Cluster Analysis Results with the Additional Dataset

Table S2. Description of the merged data set.

Exp.	Conditions (*N*)	% *F*	M Age	Reference
1	Control (53)	73.6	24.5	Grynberg, unpublished data
2	Oxytocin (47), Placebo (46)	0.0	22.6	[6]
3	Control (65)	89.2	19.6	Bukowski, Fuyimi, & Samson, unpublished data
4	Anger (26), Guilt (23)	51.0	21.2	Bukowski & Samson, unpublished data

Description of the Four Experiments

Experiment 1 can be summarized as follows: After completing the consent forms, participants completed the Positive and Negative Affects Scale [5] to assess their initial mood state. Then, participants completed the VPT task and a spatial perspective-taking task (consisting of judging whether an object is located to the left or the right from another person's spatial frame of reference). The order of these tasks was counter-balanced across participants.

Experiment 2 is described in [7] but can be summarized as follows: Participants were randomly assigned to receive either an intranasal placebo or oxytocin. Administration followed a double-blind procedure. After a 45-min break following administration, participants completed the VPT task and a mimetic desire task (described in [6]). The order of these tasks was counter-balanced across participants.

Experiment 3 can be summarized as follows: After completing the consent forms, participants completed a questionnaire about their videogame habits. Then, they completed the VPT task. After completion they played one of three types of video game meant to differ in their degree of immersion. After the video game playing they completed the VPT task again. However, only the data from before the video gaming were analysed here.

Experiment 4 can be summarized as follows: Participants were lead to believe that they played an interactive game on a computer with another person in order to induce contrasting emotional feelings, then they completed the VPT task. The participants had never met the other person face-to-face and were told they would meet her at the end of the experiment. The pictures of the human agent of the task were pictures of the other person. The interactive game was manipulated to elicit feelings of guilt (the other person lost money because of the participant) or anger (the participant lost money because of the other person who refused to share some of his money). Before the end of the experiment, participants completed an exit questionnaire assessing how they felt after the interactive game. The emotional inductions were successful in the anger condition but not the guilt condition.

Table S3. Description of the two cluster partitions based first on participants' difficulty in considering another person's differing perspective (Single-dim) and, secondly, on both the difficulty in handling conflicting perspectives (Conflict) and the attentional focus on the other person's perspective relative to the self-perspective (Focus).

Source Variables	Conflict and Focus Indexes			
Cluster #	1	2	3	4
Label	Flexible	Non-flexible	Altercentric	Egocentric
N (% total)	57 (21.9%)	43 (16.5%)	73 (28.1%)	87 (33.5%)
Female %	55.4%	62.7%	59.4%	61.9%
Conflict M (SD)	0.009 (0.033)	0.182 (0.044)	0.110 (0.044)	0.087 (0.026)
Focus M (SD)	0.001 (0.084)	−0.078 (0.062)	0.047 (0.035)	−0.044 (0.035)
Single-dim. M (SD)	1.04 (0.114)	1.426 (0.074)	1.07 (0.074)	1.19 (0.083)

Section 4: Regression Analysis Results Including Interaction Term to Predict IRI-PT

Entering the Focus index X Conflict index interaction in addition to the Conflict and the Focus indexes yielded results similar to those obtained without the interaction term (Model: $R^2 = 0.034$, F (2, 279) = 3.295, $p = 0.021$; Conflict index: $\beta = 0.038$, t (279) < 1, $p = 0.564$; Focus index: $\beta = 0.176$, t (282) = 2.881, $p = 0.004$). Most notably, the Consistency index X Perspective index interaction was not significant in predicting perspective-taking propensity ($\beta = 0.041$, t (279) < 1, $p = 0.546$).

Section 5: Two-Dimensional Cluster Analysis Results with Control Samples Only (i.e., the Samples in Which There Was No Emotional Induction)

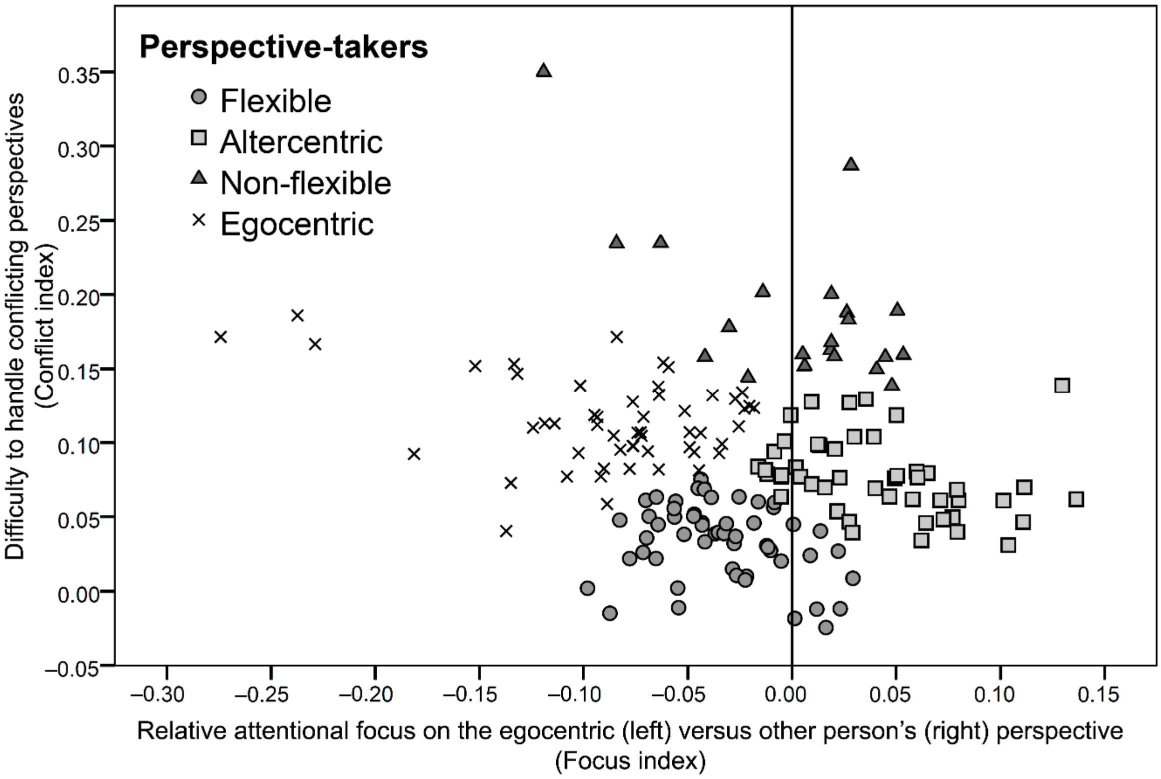

Figure S1. Two-dimensional clustering of participants allocated to the control conditions only, based on their difficulty in handling conflicting perspectives (Conflict index) and their relative attentional focus on the other person's perspective versus the self-perspective (Focus index).

References

1. Bukowski, H.; Samson, D. Can Emotions Influence Level-1 Visual Perspective Taking? *Cogn.Neurosci.* **2016**, *7*, 182–191.
2. Bukowski, H. What Influences Perspective Taking? A Dynamic and Multidimensional Approach. Ph.D. Thesis, Université catholique de Louvain (UCL), Louvain-La-Neuve, Belgium, 2014.
3. Converse, B.A.; Lin, S.; Keysar, B.; Epley, N. In the Mood to Get over Yourself: Mood Affects Theory-of-Mind Use. *Emotion* **2008**, *8*, 725–730.
4. Bukowski, H.; Hietanen, J.K.; Samson, D. From Gaze Cueing to Perspective Taking: Revisiting the Claim That We Automatically Compute Where or What Other People Are Looking at. *Vis. Cogn.* **2015**, *23*, 1020–1042.
5. Watson, D.; Clark, L.A.; Tellegen, A. Development and Validation of Brief Measures of Positive and Negative Affect: The Panas Scales. *J. Personal. Soc. Psychol.* **1988**, *54*, 1063–1070.
6. Treinen, E.; Corneille, O.; Luypaert, G. L-Eye to Me: The Combined Role of Need for Cognition and Facial Trustworthiness in Mimetic Desires. *Cognition* **2012**, *122*, 247–251.
7. Lane, A.; Mikolajczak, M.; Treinen, E.; Samson, D.; Corneille, O.; de Timary, P.; Luminet, O. Failed Replication of Oxytocin Effects on Trust: The Envelope Task Case. *PLoS ONE* **2015**, *10*, e0137000.
